# Vascular Phenotyping Across the CKM Syndrome: Arterial Stiffness and Endothelial Function Testing, Prognostic Value and Clinical Implementation

**DOI:** 10.3390/diagnostics16203273

**Published:** 2026-10-09

**Authors:** Cezara-Andreea Gerdanovics, Teodora-Gabriela Alexescu, Cezar Login, Olga Hilda Orășan, Brîndușa Tiperciuc, Anamaria-Vlăduța Tomoiagă, George Ciulei, Paul-Alexandru Milea, Dan-Claudiu Măgureanu, Luiza Maria Hiceag, Alexandra-Cătălina Andronescu, Alexandru Gerdanovics, Mircea Vasile Milaciu, Mirela-Georgiana Perne, Adela-Viviana Sitar Tăut, Angela Cozma

**Affiliations:** 1 4th Department of Internal Medicine, Faculty of Medicine, “Iuliu Hațieganu” University of Medicine and Pharmacy, 400015 Cluj-Napoca, Romaniateodora.alexescu@umfcluj.ro (T.-G.A.); vladutat@yahoo.com (A.-V.T.);; 2 Department of Physiology, “Iuliu Haţieganu” University of Medicine and Pharmacy, 400012 Cluj-Napoca, Romania; 3 Department of Pharmaceutical Chemistry, “Iuliu Haţieganu” University of Medicine and Pharmacy, 400012 Cluj-Napoca, Romania; 4 The Oncology Institute “Prof. Dr. Ion Chiricută”, 400015 Cluj-Napoca, Romania; 5 Pharmacology, Toxicology and Clinical Pharmacology, Department of Morphofunctional Sciences, “Iuliu Hațieganu” University of Medicine and Pharmacy, 400012 Cluj-Napoca, Romania; 6 Municipal Hospital Aiud, 515200 Aiud, Romania; 7 Faculty of Medicine, “Iuliu Hațieganu” University of Medicine and Pharmacy, 400347 Cluj-Napoca, Romania; 8 Department of Neurology, “Iuliu Hațieganu” University of Medicine and Pharmacy, 400012 Cluj-Napoca, Romania

**Keywords:** cardiovascular-kidney-metabolic syndrome, vascular phenotyping, arterial stiffness, endothelial dysfunction, pulse wave velocity, flow-mediated dilation, coronary artery calcium, cardiovascular risk stratification

## Abstract

Cardiovascular–kidney–metabolic (CKM) syndrome represents a continuum of metabolic, renal, and cardiovascular abnormalities in which vascular dysfunction may develop before clinically overt cardiovascular disease. Endothelial dysfunction, arterial stiffness, microvascular abnormalities, and structural vascular damage may therefore provide complementary information regarding vascular injury across CKM stages. This narrative review synthesizes current evidence on the pathophysiological basis, non-invasive assessment, prognostic value, and clinical implementation of vascular phenotyping across the CKM spectrum, with particular emphasis on arterial stiffness and endothelial function. The principal approaches discussed include carotid–femoral pulse wave velocity (cfPWV), brachial–ankle pulse wave velocity, flow-mediated dilation (FMD), peripheral arterial tonometry, microvascular techniques, and structural markers such as coronary artery calcium (CAC) and carotid plaque, considered in the context of contemporary CKM staging and PREVENT-based risk assessment. Among functional vascular measures, cfPWV remains the best-validated non-invasive marker of aortic stiffness and is independently associated with cardiovascular and renal outcomes, whereas FMD provides complementary information on conduit-artery endothelial function but is limited by technical requirements and inconsistent incremental predictive value. Microvascular and glycocalyx-based techniques remain less standardized, while structural phenotyping, particularly CAC and carotid plaque assessment, has stronger evidence for risk reclassification in selected populations. Vascular phenotyping should therefore be regarded as a selective, risk-adapted strategy rather than a universal screening approach. Its greatest potential may lie in integrating vascular measures with CKM stage, PREVENT estimates, kidney and cardiac biomarkers, and structural imaging to identify disproportionate or subclinical vascular injury, particularly during progression from CKM stage 2 to stage 3. Prospective studies are needed to determine whether such multimodal phenotyping improves therapeutic decision-making and cardiovascular and kidney outcomes.

## 1. Introduction

Cardiovascular–kidney–metabolic (CKM) syndrome encompasses a continuum of interconnected metabolic, renal, and cardiovascular abnormalities associated with progressive multiorgan involvement and increasing cardiovascular risk [1]. Within this framework, vascular alterations may develop before clinically overt cardiovascular or kidney disease, making the vasculature a potentially informative target for identifying subclinical disease and characterizing risk across different stages of the CKM spectrum [1,2,3].

Vascular phenotyping provides a means of capturing complementary aspects of subclinical cardiovascular injury. These approaches can be broadly organized according to the vascular domain assessed. Large-artery stiffness and central hemodynamics can be characterized using carotid–femoral pulse wave velocity (cfPWV), brachial–ankle pulse wave velocity (baPWV), estimated pulse wave velocity (ePWV), augmentation index, and central blood pressure. Endothelial and microvascular function can be evaluated using flow-mediated dilation (FMD), peripheral arterial tonometry with reactive hyperemia index, laser Doppler or laser speckle techniques, and emerging approaches assessing endothelial glycocalyx integrity. Structural vascular phenotyping includes carotid and femoral ultrasound for intima–media thickness and plaque burden, coronary artery calcium (CAC) scoring, and additional imaging-based markers of subclinical vascular disease. These techniques interrogate distinct but interrelated components of vascular biology, ranging from potentially reversible functional abnormalities to cumulative arterial remodeling and established atherosclerotic burden, and should therefore be regarded as complementary rather than interchangeable [3,4,5,6]. The cumulative burden of cardiometabolic abnormalities may also be relevant to the development of subclinical vascular injury. In a recent cross-sectional study of individuals without clinically overt atherosclerotic cardiovascular disease, a greater number of metabolic syndrome components, irrespective of their specific constellation, was associated in univariate analyses with higher carotid, common femoral, and superficial femoral intima–media thickness, as well as with higher cfPWV, central pulse pressure, and end-systolic pressure. However, most of these associations were attenuated after adjustment for age, sex, and body mass index, highlighting both the potential relationship between cumulative metabolic burden and vascular dysfunction and the substantial influence of conventional demographic and adiposity-related determinants [6].

Despite the growing evidence supporting vascular phenotyping, its translation into routine CKM assessment remains uncertain. Available techniques differ in standardization, reproducibility, operator dependence, accessibility, and prognostic evidence, and most are not currently incorporated into formal CKM staging or routine risk-assessment algorithms [1,2]. Defining which vascular tests provide clinically meaningful information, in whom they should be applied, and whether they improve risk stratification beyond established clinical and laboratory markers therefore remains an important challenge.

Accordingly, this review examines vascular phenotyping across the CKM syndrome, with particular emphasis on endothelial function and arterial stiffness. We summarize their pathophysiological basis, available non-invasive assessment methods, prognostic significance and practical limitations, and discuss their potential integration into CKM risk stratification and clinical practice.

## 2. Narrative Review Methodology

This article was designed as a narrative rather than a systematic review. The literature was identified through searches of PubMed/MEDLINE, Scopus and Web of Science, complemented by manual screening of the reference lists of relevant reviews, consensus documents, and clinical guidelines. Searches included combinations of terms related to “cardiovascular-kidney-metabolic syndrome”, “CKM syndrome”, “vascular phenotyping”, “arterial stiffness”, “pulse wave velocity”, “endothelial dysfunction”, “flow-mediated dilation”, “microvascular dysfunction”, “glycocalyx”, “carotid plaque”, “coronary artery calcium”, “vascular calcification”, “cardiovascular risk”, and “kidney outcomes”.

Literature published up to July 2026 was considered. Priority was given to contemporary clinical guidelines, consensus statements, systematic reviews and meta-analyses, large prospective cohort studies and studies evaluating prognostic or clinical utility of vascular phenotyping techniques. Seminal methodological studies and older landmark reports were retained when necessary to describe the development, validation or interpretation of specific vascular measurements.

Studies were selected on the basis of relevance to vascular structure, function, prognosis, and clinical implementation across the CKM syndrome. Because the aim was interpretative and clinically integrative rather than systematic evidence synthesis, no formal PRISMA-based study-selection process, risk-of-bias assessment or quantitative meta-analysis was performed. The stage-specific implementation framework presented in this Review was developed by integrating the identified evidence with contemporary CKM guidelines and risk-assessment recommendations.

## 3. Pathophysiological Rationale for Vascular Phenotyping in CKM

Vascular dysfunction in CKM syndrome is heterogeneous and can be differentiated according to the vascular compartment involved, the dominant pathophysiological mechanism, and the extent to which abnormalities are predominantly functional or structural. Large-artery phenotypes primarily reflect altered arterial wall mechanics, remodeling, and central hemodynamic load, whereas endothelial phenotypes capture impaired vasomotor, antithrombotic, anti-inflammatory, and barrier functions. Microvascular phenotypes involve abnormalities in tissue perfusion, capillary structure, and endothelial–pericyte interactions, while structural vascular phenotypes reflect more advanced or cumulative injury such as intimal thickening, atherosclerotic plaque formation, calcification, and arterial remodeling. These manifestations are biologically interconnected and may coexist within the same patient, but they are not interchangeable because they reflect different stages and dimensions of vascular injury across the CKM syndrome.

Vascular phenotyping in CKM syndrome is supported by its underlying pathophysiology, as insulin resistance and adipose tissue dysfunction promote early vascular alterations through interconnected inflammatory, neurohormonal, and hemodynamic mechanisms. These vascular changes may precede clinically evident cardiovascular and renal disease, supporting the assessment of subclinical vascular dysfunction as an early indicator of systemic and multiorgan risk [1,7].

### 3.1. Metabolic Drivers of Endothelial Dysfunction and Vascular Remodeling

Adipose tissue dysfunction, particularly visceral adiposity, is a key component of CKM syndrome [1]. Maladaptive expansion promotes adipocyte hypertrophy, hypoxia, immune-cell infiltration, inflammation, and insulin resistance [8], together with reduced adiponectin and adipsin and increased resistin and RBP4 [9]. Increased free fatty acids, ROS, ectopic lipid deposition, lipotoxicity, and cellular stress further amplify metabolic dysfunction [8], defining adiposopathy and promoting systemic cardiometabolic disease [10]. Perivascular adipose tissue (PVAT), normally vasodilatory, anticontractile, and anti-inflammatory [11], becomes proinflammatory and pro-oxidative in obesity and insulin resistance [12,13,14]. Cytokines, adipokines, and ROS then promote endothelial activation, leukocyte recruitment, vascular smooth muscle cell proliferation, and vascular remodeling [12,13], supporting an “outside-in” model of vascular inflammation [12].

**Figure 1:**
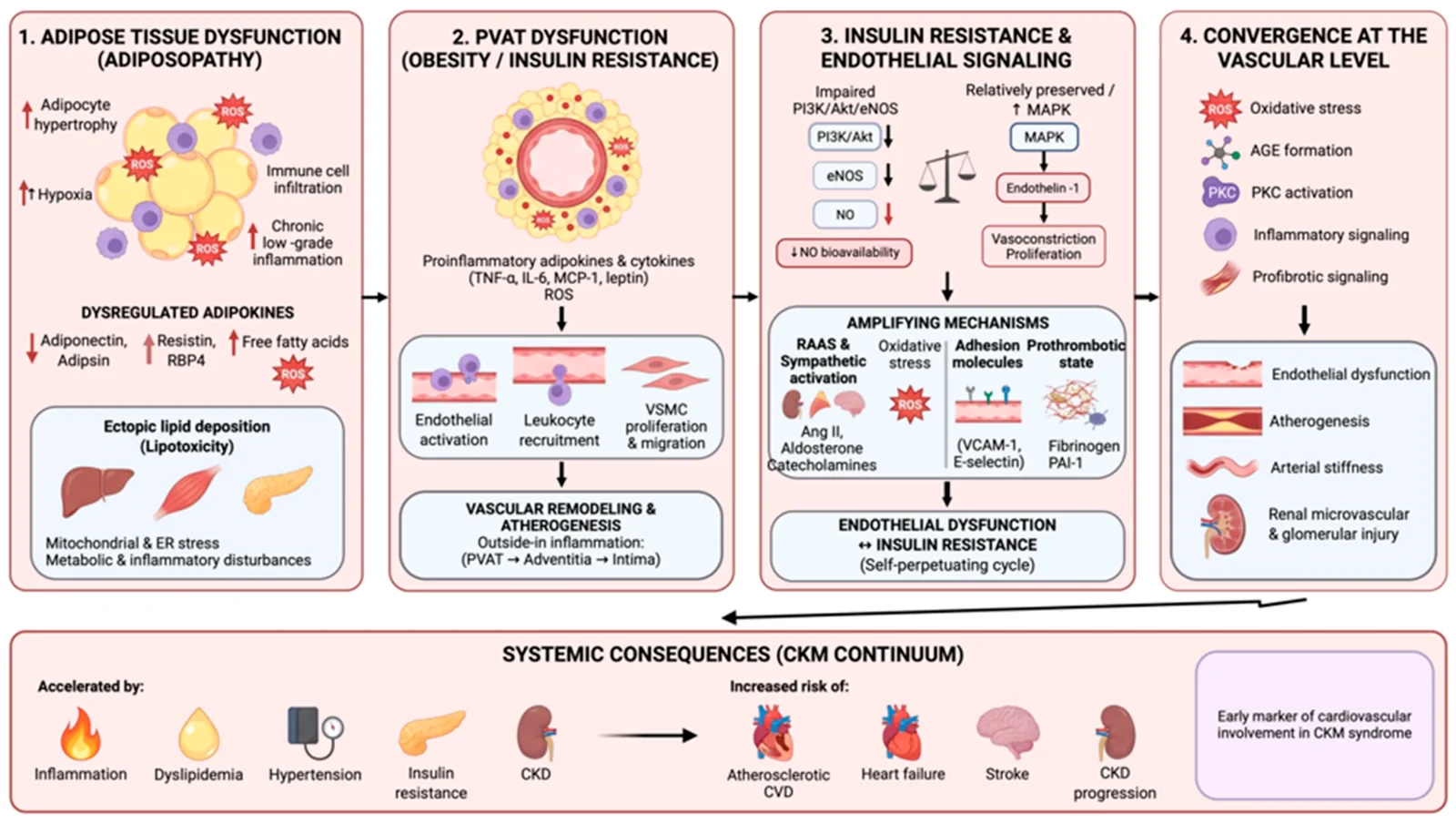
Pathophysiological interplay between adipose tissue dysfunction, insulin resistance and vascular remodeling in CKM syndrome. (1) Adipose tissue dysfunction is characterized by adipocyte hypertrophy, inflammation, adipokine imbalance, and lipotoxicity. (2) Dysfunctional perivascular adipose tissue (PVAT) promotes local vascular inflammation and remodeling. (3) Insulin resistance disrupts endothelial signaling, reducing nitric oxide (NO) bioavailability and favoring vasoconstrictive, pro-oxidative, and prothrombotic pathways. (4) These mechanisms converge at the vascular level, leading to endothelial dysfunction, atherogenesis, arterial stiffness, and renal microvascular injury. (5) Progressive vascular damage contributes to cardiovascular and kidney complications across the CKM continuum. Created in BioRender. Vladuta, T. (2026) https://BioRender.com/alsz11p.

Insulin resistance also disrupts endothelial signaling. PI3K/Akt/eNOS normally promotes nitric oxide (NO)-dependent vasodilation, whereas MAPK signaling favors endothelin-1 production and proliferation [15,16]. Selective impairment of PI3K/Akt/eNOS with preserved MAPK activity reduces NO bioavailability and promotes vasoconstriction and vascular smooth muscle proliferation [15,17]. RAAS and sympathetic activation, increased VCAM-1 and E-selectin expression [15], and reduced NO-mediated antiplatelet activity with increased fibrinogen and PAI-1 [15] further create a pro-oxidative, inflammatory, and prothrombotic environment. Impaired NO-dependent vasodilation may also reduce skeletal-muscle glucose delivery, reinforcing insulin resistance [17]. Ultimately, oxidative stress, advanced glycation end products, protein kinase C activation, and inflammatory/profibrotic signaling [1] converge to promote endothelial dysfunction, atherogenesis, arterial stiffness, and renal microvascular injury [1], as summarized in Figure 1.

### 3.2. Arterial Stiffness, Vascular Remodeling and Calcification

Large-artery stiffening and kidney dysfunction are reciprocally interconnected across the CKM continuum, but vascular remodeling may precede clinically evident kidney disease. Loss of aortic elasticity increases transmission of pulsatile energy to the high-flow, low-resistance renal microcirculation [3,18], promoting glomerular injury, albuminuria, and nephron loss [3,18]. Increased left ventricular afterload and impaired coronary perfusion additionally link arterial stiffening to cardiovascular injury across the CKM continuum [3,18,19].

As kidney function declines, the vascular wall is increasingly exposed to disturbed mineral metabolism, uremic toxins, oxidative and inflammatory stress, and neurohormonal activation, further promoting arterial remodeling and loss of compliance [3,18,19]. CKD–mineral and bone disorder, particularly phosphate retention, favors osteogenic transformation of vascular smooth muscle cells and medial calcium–phosphate deposition [3,19]. Uremic solutes such as indoxyl sulfate, p-cresyl sulfate, and trimethylamine N-oxide, together with uric acid, asymmetric dimethylarginine, and endothelin-1, further promote endothelial dysfunction, oxidative stress, and vascular calcification [19,20].

Oxidative and inflammatory pathways reduce nitric oxide bioavailability and promote vascular smooth muscle and extracellular matrix remodeling [3,21]. These effects are amplified by renin–angiotensin–aldosterone and sympathetic activation and by sodium and volume dysregulation, increasing vascular tone, fibrosis, arterial stiffness, and cardiac afterload [3,18]. Progressive nephron loss then further aggravates mineral dysregulation, uremic toxin retention, oxidative stress, and neurohormonal activation, creating a self-amplifying cardiorenal cycle.

### 3.3. Endothelial Dysfunction as an Early Vascular Phenotype

Endothelial dysfunction is an early and integrative manifestation of vascular injury across the CKM continuum and may precede detectable cardiovascular or renal structural disease [1,6]. Loss of vasodilatory, anti-inflammatory, antithrombotic, and barrier-protective functions promotes progressive vascular and target-organ injury [1].

Albuminuria illustrates the systemic nature of endothelial dysfunction. Beyond reflecting glomerular injury, increased urinary albumin excretion may indicate generalized impairment of endothelial barrier integrity [1,22], consistent with the Steno hypothesis [22]. The association of increasing urinary albumin-to-creatinine ratio (UACR) with both cardiovascular and renal outcomes further links glomerular and systemic vascular injury [1,22].

CKD may further aggravate endothelial dysfunction through glycocalyx disruption, hypoxic stress, cellular senescence, and endothelial-to-mesenchymal transition, progressively impairing barrier function and reparative capacity [7,21]. Endothelial dysfunction may therefore represent an intermediate phenotype linking cardiometabolic risk to overt cardiovascular and kidney disease, although its incremental clinical utility beyond established CKM markers such as eGFR and UACR requires prospective validation [1,6].

### 3.4. Microvascular Versus Macrovascular Injury

Microvascular dysfunction may precede clinically detectable macrovascular disease in cardiometabolic and kidney disorders [23]. Early abnormalities are predominantly functional and potentially reversible [24], whereas prolonged metabolic and hemodynamic stress promotes structural microangiopathy characterized by basement-membrane thickening, capillary rarefaction, and pericyte loss [25]. Beyond retinal, renal, and peripheral nerve involvement, microvascular dysfunction affects the myocardium, skeletal muscle, and cerebral circulation and has been linked to HFpEF, impaired muscle function, and cognitive decline [25].

Macrovascular injury reflects progressive atherosclerosis, calcification, and loss of arterial compliance, with CKD markedly increasing vascular calcification and peripheral arterial disease burden [1]. Large-artery stiffness is closely related to declining renal function [3,20,26] and increases cardiac afterload while potentially impairing diastolic coronary perfusion [20].

Micro- and macrovascular injury interact bidirectionally. Increased central arterial stiffness enhances transmission of pulsatile energy to low-resistance microvascular beds, particularly the kidney, brain, and heart, promoting endothelial injury and target-organ damage [1,3]. Conversely, microvascular dysfunction may facilitate macrovascular remodeling through tissue hypoxia, impaired perfusion, and vasa vasorum abnormalities [1].

Thus, functional microvascular and endothelial abnormalities may identify earlier vascular injury, whereas large-artery measures reflect more cumulative damage [3,23,24,25]. Integrating albuminuria and endothelial/microvascular measures with cfPWV, arterial calcification, and atherosclerotic burden may provide a more comprehensive CKM vascular phenotype, although its incremental clinical value requires prospective validation [2,3,26].

## 4. Arterial Stiffness Phenotype

Large-artery stiffening, particularly of the aorta, is a hallmark of arteriosclerosis and is mechanistically distinct from plaque-driven atherosclerosis. It reflects the interaction of structural remodeling and potentially reversible functional changes, with disease-specific contributions from hypertension, diabetes, metabolic disease, CKD, aging, and hemodynamic factors [27,28]. Aortic stiffness is also independently associated with cardiovascular risk, supporting its role as a clinically relevant vascular phenotype rather than merely a consequence of aging or hypertension [29].

### 4.1. Carotid–Femoral Pulse Wave Velocity

Aortic pulse wave velocity represents the reference concept for assessing central arterial stiffness, while carotid–femoral pulse wave velocity (cfPWV) is the most extensively validated and clinically accessible non-invasive surrogate [30,31,32,33]. cfPWV is derived from pulse transit time and an estimated carotid–femoral travel distance, and standardized acquisition remains essential because differences in path-length measurement can systematically influence absolute values [34,35,36,37,38]. The commonly used threshold of approximately 10 m/s therefore depends on standardized distance methodology and should not be interpreted independently of the measurement protocol [36,37,38]. From a biological perspective, increased cfPWV reflects reduced aortic compliance resulting from both functional changes in vascular tone and cumulative structural remodeling of the arterial wall, including elastin fragmentation, collagen deposition, fibrosis, calcification, and vascular smooth-muscle alterations. It therefore provides an integrated measure of large-artery vascular aging rather than a direct measure of atherosclerotic plaque burden. Its prognostic value exceeds that of several other hemodynamic indices in population studies [34], and progressively increased cfPWV may identify a vascular remodeling phenotype disproportionate to chronological age or conventional risk-factor burden. Within CKM syndrome, this interpretation is particularly relevant because metabolic dysfunction, hypertension, diabetes, and CKD converge on arterial remodeling through partly overlapping mechanisms. Thus, cfPWV is most informative when used to characterize cumulative large-artery injury and accelerated vascular aging within the broader CKM phenotype rather than as an isolated diagnostic threshold [32,39].

### 4.2. Brachial–Ankle and Estimated PWV

Brachial–ankle PWV (baPWV) offers greater scalability through simultaneous four-limb cuff measurements and height-based path-length estimation [40]. Unlike cfPWV, it integrates both central elastic and peripheral muscular arterial properties and is therefore influenced by peripheral vascular tone and cannot be considered interchangeable with cfPWV [41]. Both measures are associated with hypertensive target-organ damage, although their associations differ across organ systems [42,43]. baPWV also has established prognostic value, with higher values associated with cardiovascular events and mortality [40,44], and its simplicity has facilitated broad use in Asian vascular-aging programs [45].

Estimated PWV (ePWV) requires no direct vascular measurement and is calculated from age and mean arterial pressure using validated equations [46]. It has independently predicted cardiovascular outcomes, including in SPRINT, with performance approaching measured cfPWV in some populations [46,47]. Comparisons with baPWV suggest partly complementary prognostic information, although measured baPWV may provide greater discrimination in some cohorts [48]. Because ePWV is derived largely from age and blood pressure, however, it cannot identify individual deviations from expected vascular stiffness and is therefore less suitable for individualized phenotyping [49]. Thus, cfPWV provides greater physiological specificity, whereas baPWV and ePWV offer progressively greater scalability at the expense of direct characterization.

### 4.3. Augmentation Index and Wave Reflection

Arterial stiffness also alters pulse-wave morphology. The central pressure waveform reflects the interaction of the forward wave generated by ventricular ejection with waves returning from peripheral sites of impedance mismatch [50,51]. With increasing arterial stiffness, faster wave propagation causes reflected waves to return earlier, augmenting late-systolic central pressure rather than supporting diastolic pressure [52,53].

The augmentation index (AIx) quantifies the contribution of wave reflection to pulse pressure and is commonly derived from carotid or radial waveforms [39]. However, AIx is strongly affected by heart rate, height, age, and sex and therefore does not represent a pure measure of aortic stiffness [39]. Rather, it integrates arterial properties, peripheral wave reflection, and vascular resistance and is better regarded as a marker of ventricular–arterial load [39]. Despite these dependencies, AIx and related central hemodynamic indices have demonstrated independent associations with cardiovascular events and mortality [54] and provide information complementary to PWV.

### 4.4. Central Blood Pressure and Pulse Pressure Amplification

Earlier return of reflected waves with arterial stiffening increases central systolic pressure and reduces the normal amplification of pulse pressure from the aorta to the brachial circulation [55,56]. Central pressure may therefore better represent the hemodynamic load experienced by the heart, brain, and kidneys than brachial pressure alone. In the Strong Heart Study, central pressure was more strongly associated with vascular hypertrophy, atherosclerosis, and cardiovascular events than brachial pressure [57]. Similarly, the CAFE substudy demonstrated that antihypertensive regimens producing comparable brachial blood-pressure reductions could produce substantially different central aortic pressures and cardiovascular outcomes [58,59], supporting the prognostic relevance of central hemodynamics [28].

Antihypertensive classes may also differ in their effects on central pressure, with vasodilating agents generally producing greater reductions than conventional beta-blockade despite similar brachial pressure lowering [1,58,59,60,61]. Nevertheless, whether routine central-pressure-guided treatment improves outcomes beyond conventional blood-pressure management remains less firmly established, and these indices should primarily be regarded as complementary tools for vascular phenotyping and risk characterization [54].

Overall, cfPWV, baPWV/ePWV, AIx and central blood pressure characterize distinct but overlapping dimensions of the arterial stiffness phenotype—wave propagation, wave reflection, and resulting hemodynamic load. Their clinical interpretation should therefore emphasize complementary information rather than interchangeable measurements and should be integrated with the broader CKM phenotype.

### 4.5. Clinical Interpretation Across CKM Stages

The AHA CKM framework spans stage 0 to stage 4 and integrates metabolic, kidney, and cardiovascular disease progression [35,36]. Arterial stiffness may complement this framework as a marker of vascular aging, with vascular-aging indices associated with both CKM stage and cardiovascular risk [62,63]. At stages 0–1, abnormalities may remain subtle, with AIx or divergence between measured and estimated PWV potentially preceding overt metabolic disease [49]. At stage 2, cfPWV elevation and altered wave reflection become more apparent and associate with target-organ damage [42], whereas stages 3–4 are characterized by progressively greater central hemodynamic burden and vascular remodeling [42,55], including advanced arterial stiffening in established disease [42].

Because individual indices capture different aspects of vascular aging, no single stiffness measure is optimal across the entire CKM spectrum [39]. Arterial stiffness should therefore be regarded as a complementary vascular phenotype with independent prognostic relevance [27,28], rather than as a formal CKM staging criterion. Its mechanical information is complemented by endothelial dysfunction, which may emerge earlier during metabolic and hemodynamic stress [63].

## 5. Endothelial Function Phenotype

Endothelial function integrates the cumulative vascular effects of cardiovascular risk factors and may improve when adverse exposures are modified [64]. However, endothelial dysfunction is heterogeneous: conduit-artery and microvascular measurements interrogate related but distinct vascular compartments [64,65]. Most tests assess vasodilator responses to reactive hyperemia or vasoactive stimuli, frequently through nitric oxide (NO)-dependent mechanisms [64,66]. Because methods differ in vascular bed, stimulus, invasiveness, and reproducibility, no single technique captures the entire endothelial phenotype [64]. Invasive coronary or forearm assessments remain reference research approaches but are limited by cost and procedural risk [64,65].

### 5.1. Flow-Mediated Dilation

Flow-mediated dilation (FMD) is the most established non-invasive method for assessing conduit-artery endothelial function. It quantifies the change in brachial artery diameter during reactive hyperemia after transient forearm occlusion and is predominantly, although not exclusively, nitric oxide dependent [4,67]. Interpretation requires standardized participant preparation, vascular imaging, and analysis because FMD is influenced by age, body size, smoking, baseline arterial diameter, hemodynamic conditions, and methodological variability [4,67,68,69,70,71,72,73,74]. Endothelium-independent dilation after nitroglycerin may help distinguish impaired endothelial signaling from abnormalities of vascular smooth-muscle responsiveness [70]. Biologically, impaired FMD primarily reflects an early functional component of the vascular remodeling continuum, characterized by reduced nitric oxide bioavailability and altered endothelial vasomotor responsiveness. Unlike cfPWV, which increasingly reflects cumulative large-artery mechanical and structural remodeling, FMD may identify vascular dysfunction before advanced structural disease becomes apparent. Lower FMD has been associated with subclinical vascular disease and future cardiovascular events [4,6,67], although heterogeneous reference values and modest incremental predictive values limit its use as an isolated risk-reclassification tool [68,69]. Within CKM syndrome, FMD may therefore be most informative as a complementary marker of early endothelial involvement, particularly when interpreted together with arterial stiffness and structural measures of vascular injury rather than as a stand-alone diagnostic endpoint.

### 5.2. Peripheral Arterial Tonometry and Reactive Hyperemia Index

Peripheral arterial tonometry (PAT) provides a largely operator-independent assessment of digital microvascular function [75,76]. Finger plethysmography records pulse-wave amplitude before and after brachial occlusion, from which the reactive hyperemia index (RHI) is calculated after normalization to the contralateral arm [76,77]; logarithmic transformation is also commonly used [77]. Reproducibility is acceptable under standardized testing conditions [66].

PAT responses appear partly NO-dependent, and proposed abnormal thresholds include RH-PAT < 1.35 and EndoPAT RHI ≤ 1.67 [77,78,79]. Lower RHI has predicted adverse cardiovascular events in selected cohorts [78], including after adjustment for conventional risk scores [80] and in HFpEF [81]. However, population data are less consistent: PAT measures were not associated with composite cardiovascular events in Framingham, although higher PAT response was associated with lower ischemic stroke risk [82]. Questions also remain regarding its specificity for NO-dependent endothelial function [77]. PAT/RHI and FMD should therefore be considered complementary rather than interchangeable measures [77,82].

### 5.3. Laser-Based Assessment of Skin Microvascular Reactivity

The skin provides an accessible model of systemic microvascular function [83,84]. Laser Doppler and laser speckle techniques quantify red-blood-cell-derived perfusion signals and can be combined with reactive hyperemia, local heating, or pharmacological stimulation [85,86]. Single-point laser Doppler flowmetry offers high temporal resolution but samples a small heterogeneous tissue volume and has limited reproducibility [82,83,84,85,86]. Laser Doppler perfusion imaging improves spatial coverage but has lower temporal resolution [86], whereas laser speckle contrast imaging (LSCI) combines rapid, non-contact imaging with comparatively high reproducibility [83] and lower intersubject variability than LDF [87].

Endothelium-dependent and -independent responses can be explored using acetylcholine and sodium nitroprusside [83,87], while local heating [87], post-occlusive reactive hyperemia [84,87], and spectral analysis [84] provide additional functional information. Nevertheless, absolute values derived from different laser techniques are not interchangeable [85], limiting standardized clinical interpretation.

### 5.4. Endothelial Glycocalyx and Capillaroscopy

The endothelial glycocalyx regulates permeability, mechanotransduction, immune-cell interactions, and vascular tone [88,89], and its disruption has been implicated in diabetes, atherosclerosis, ischemic disease, and other vascular disorders [26,28]. Sublingual sidestream dark-field (SDF) videomicroscopy with automated analysis provides a non-invasive estimate of glycocalyx integrity [89,90,91], most commonly through the perfused boundary region (PBR), where higher values indicate greater red-blood-cell penetration and impaired barrier properties [89,90]. PBR has also been associated with impaired microvascular perfusion [80].

Although early studies suggested favorable reproducibility, subsequent work demonstrated operator dependence and variability [79,80,81,82]. Higher PBR has been associated with arterial stiffness and abnormalities of coronary and myocardial function in hypertension [89], while additional automated indices can characterize microvascular density and RBC filling [91]. However, large community studies found no consistent independent association between PBR and prevalent cardiovascular disease [92], and substantial overlap between patients with and without obstructive CAD limits discrimination [91]. Glycocalyx imaging should therefore currently be regarded as an exploratory rather than established clinical phenotype.

### 5.5. Practical Limitations of Endothelial Testing

Endothelial techniques assess distinct vascular beds and biological mechanisms and are therefore not interchangeable [64]. Invasive testing remains restricted largely to research because of cost and procedural risk [64,65]. FMD has the strongest evidence among non-invasive methods but remains technically demanding and operator-dependent [4,65], requiring strict control of participant preparation and acquisition conditions [67,72,74,75]. Moreover, FMD may be influenced by non-endothelial hemodynamic factors such as transmural pressure, arterial stiffness, and central blood pressure [93].

PAT is simpler and less operator-dependent, but its NO specificity and prognostic consistency remain uncertain [20,77], with additional variability related to timing and repeated measurements [94] and heterogeneous abnormality thresholds [76,79,80]. Laser-based methods remain sensitive to spatial heterogeneity, motion, temperature, and differences in acquisition and provocation protocols [83,84,85,86]. Similarly, SDF-derived glycocalyx measures show variable reproducibility and limited independent cardiovascular discrimination [91,92].

Accordingly, standardized methodology, particularly for FMD, and population-appropriate reference values remain essential [4,64,68]. Further validation is required before newer endothelial techniques can be incorporated into routine CKM assessment [64].

## 6. Structural Vascular Phenotype

Structural vascular phenotyping captures the cumulative anatomical consequences of CKM-related vascular injury, including intimal thickening, atherosclerotic plaque, calcification, and remodeling [95]. Within the AHA CKM framework, imaging-detected subclinical atherosclerosis and coronary artery calcium (CAC) contribute to the identification of stage 3 disease [61].

### 6.1. Carotid Intima–Media Thickness and Plaque Burden

Carotid intima–media thickness (cIMT) has long been used as a surrogate of subclinical vascular disease, but its incremental predictive value is limited. Although increasing cIMT is associated with myocardial infarction and stroke, adding it to conventional risk models produced only modest reclassification in meta-analysis [96]. Similarly, in MESA, cIMT provided substantially less incremental discrimination than CAC [97]. This partly reflects the fact that plaque-free cIMT represents pressure- and age-related medial hypertrophy as well as atherosclerosis, while methodological heterogeneity further limits interpretation [98].

Accordingly, emphasis has shifted toward plaque detection and quantification. Carotid plaque predicts coronary events more accurately than cIMT, while plaque burden provides additional information beyond plaque presence alone [98,99]. Multiterritory plaque assessment may further improve risk prediction [99]. Contemporary cohorts have demonstrated incremental prognostic value when carotid plaque or plaque burden is added to SCORE2 [100,101], while the P-SONAR studies support weighted carotid–femoral plaque burden as a potentially informative marker even among individuals classified as low risk by conventional algorithms [102,103,104,105,106]. Carotid and femoral ultrasound is inexpensive, radiation-free, and clinically accessible, and the 2024 ESC hypertension guideline recognizes plaque assessment, CAC, and PWV as potential risk modifiers when treatment decisions remain uncertain [107].

### 6.2. Coronary Artery Calcium Score

CAC measured by non-contrast ECG-gated CT is the structural marker with the strongest evidence for ASCVD risk reclassification. In MESA, increasing CAC strongly predicted coronary events and provided the largest improvement in discrimination among several candidate markers [108]. In the Rotterdam Study, CAC also substantially improved risk classification, particularly in intermediate-risk individuals, outperforming cIMT, PWV, C-reactive protein, and natriuretic peptide [109].

Its relevance extends directly to CKM populations. In individuals with diabetes or metabolic syndrome, CAC improved long-term risk classification, while CAC = 0 identified subgroups with relatively low event rates [110]. In CKD, CAC becomes increasingly prevalent with declining kidney function and predicts cardiovascular events and mortality beyond eGFR and albuminuria [110]. Calcification across coronary, carotid, and thoracic aortic territories has also independently predicted cardiovascular events and death across CKD stages [111]. Interpretation in CKD and diabetes nevertheless requires caution because the Agatston score does not distinguish intimal atherosclerotic from medial calcification, which is disproportionately prevalent in these populations [112,113].

### 6.3. Retinal Vascular Imaging

The retinal circulation provides direct, non-invasive visualization of the microvasculature. Retinal vessel caliber, tortuosity, fractal characteristics, OCT, and OCT angiography have been associated with hypertension, diabetes, stroke, CKD, and early microvascular abnormalities [114]. Deep-learning analysis of fundus photographs can derive cardiovascular risk-related phenotypes [115], while retinal models such as RetiCAC have shown potential for cardiovascular-event prediction [116]. In CKD, retinal-based prediction models and AI-derived vessel measurements have also demonstrated associations with incident CKD and cardiovascular disease, although improvements over conventional models have generally been modest [117]. Retinal imaging should therefore currently be regarded as a promising complementary rather than established risk-reclassification tool.

### 6.4. Renal and Systemic Vascular Imaging

Renal vascular assessment lies between structural and functional phenotyping. The renal resistive index (RRI), derived from intrarenal Doppler flow, has been associated with kidney-function decline and mortality. An RRI ≥ 0.70 independently predicted all-cause mortality in patients with CKD stages 3–4 [117].

Systemic calcification burden provides additional structural information. Abdominal aortic calcification (AAC), measurable on radiographs, DXA or CT, predicts cardiovascular events and mortality, with particularly strong associations in CKD populations [117]. These measures may therefore complement coronary and carotid assessment when systemic or renal vascular involvement is clinically relevant.

### 6.5. Functional Versus Structural Phenotyping

Functional and structural vascular phenotypes represent interconnected rather than competing processes. PWV reflects both arterial wall composition and dynamic hemodynamic influences, endothelial dysfunction contributes to subsequent remodeling, and established calcification further increases stiffness [118]. RRI is predominantly hemodynamic, while CAC and AAC in CKD may substantially reflect medial calcification, and plaque-free cIMT partly reflects pressure-driven medial hypertrophy [117,119].

For risk prediction, structural markers—particularly CAC—generally outperform functional measures. In MESA, CAC provided substantially greater incremental discrimination than FMD [97], while in the Rotterdam Study it reclassified intermediate-risk individuals more effectively than cfPWV [106]. Nevertheless, aortic PWV independently improves cardiovascular risk prediction, with greater reclassification benefit in younger and intermediate-risk individuals [120].

Importantly, these comparisons were not stratified by CKM stage. Functional abnormalities may become detectable earlier in the CKM continuum, whereas structural plaque and calcification increasingly reflect accumulated disease burden at more advanced stages. Supporting this concept, estimated PWV has been associated with CKM stage and mortality [121]. The most appropriate clinical interpretation is therefore complementarity rather than substitution: functional measures characterize vascular dysfunction and aging, whereas structural imaging quantifies established vascular burden. Consistent with this approach, contemporary ESC guidance recognizes CAC, vascular plaque, and PWV as alternative risk modifiers selected according to clinical context, availability, cost, and shared decision-making [104]. Importantly, the vascular phenotypes summarized in Table 1 should not be interpreted as mutually exclusive categories. Rather, they represent complementary dimensions of vascular structure and function that may coexist to varying degrees within the same patient. For example, endothelial dysfunction may precede or accompany increased arterial stiffness, while structural atherosclerosis and vascular calcification may subsequently coexist with both functional abnormalities. The resulting vascular phenotype is therefore best viewed as a multidimensional composite, in which the relative contribution of endothelial, microvascular, large-artery mechanical, and structural abnormalities varies according to CKM stage, comorbidity burden, and disease duration. Accordingly, the categories presented in Table 1 are intended as an interpretative framework linking each assessment modality to the vascular domain it predominantly interrogates, rather than as discrete patient subtypes.

To facilitate comparison across modalities, Table 1 summarizes the principal vascular phenotyping techniques discussed in this review, highlighting the vascular compartment assessed, their main strengths and limitations, and their potential clinical role across the CKM spectrum.

## 7. Prognostic Value: Cardiovascular Events, Mortality and Kidney Outcomes

Vascular phenotyping may provide prognostic information beyond conventional risk factors by reflecting the cumulative vascular effects of metabolic, renal, inflammatory, and hemodynamic abnormalities. The strongest outcome evidence concerns arterial stiffness, particularly carotid–femoral pulse wave velocity (cfPWV), whereas endothelial and microvascular measures show more variable prognostic performance [122].

### 7.1. Arterial Stiffness and Cardiovascular Events

Aortic and cfPWV consistently predict cardiovascular events and mortality. In an individual-participant meta-analysis of 17,635 subjects, including 1785 cardiovascular events, each 1-SD increase in log-transformed aortic PWV remained independently associated with coronary heart disease (HR 1.23, 95% CI 1.11–1.35), stroke (HR 1.28, 95% CI 1.16–1.42), and total cardiovascular events (HR 1.30, 95% CI 1.18–1.43) after adjustment for conventional risk factors. Addition of aortic PWV improved 10-year cardiovascular risk reclassification by approximately 13% among intermediate-risk individuals [123]. Individual-participant data subsequently confirmed independent associations with coronary heart disease, stroke, and total cardiovascular events and demonstrated improved reclassification, particularly among intermediate-risk individuals [122]. More recent meta-analyses have confirmed graded associations between cfPWV and cardiovascular events and mortality [124,125].

More recently, an individual-participant meta-analysis focused on individuals with moderately elevated blood pressure reported 1279 first ASCVD events over a median follow-up of 9.9 years; each 1-SD increase in log-transformed cfPWV was associated with a 21% higher risk of ASCVD (HR 1.21, 95% CI 1.08–1.36), while addition of cfPWV increased the C-index by 0.0048 (95% CI 0.0002–0.0094) [120].

Importantly, cfPWV also improves risk discrimination in individuals with moderately elevated blood pressure, supporting its potential value when conventional risk classification remains uncertain [3,122]. Overall, cfPWV remains the best-validated non-invasive arterial-stiffness marker for cardiovascular prognosis.

### 7.2. Arterial Stiffness in Metabolic Disease and CKD

Arterial stiffening develops early in dysglycemia and type 2 diabetes and reflects interacting effects of hyperglycemia, advanced glycation, insulin resistance, endothelial dysfunction, and inflammation; it independently predicts cardiovascular outcomes in these populations [3,126]. Its prognostic importance is particularly marked in CKD, where accelerated vascular aging and medial calcification amplify pulsatile vascular load [122].

In the CRIC cohort of 2795 patients with CKD, participants in the highest cfPWV tertile (>10.3 m/s) had a 37% higher adjusted risk of end-stage kidney disease (HR 1.37, 95% CI 1.05–1.80) and a 72% higher risk of all-cause mortality (HR 1.72, 95% CI 1.24–2.38) compared with those in the lowest tertile; the association with the composite of end-stage kidney disease or 50% eGFR decline was weaker (HR 1.25, 95% CI 0.98–1.58) [4]. In a large NHANES CKM cohort, higher estimated PWV was associated with both all-cause and cardiovascular mortality, with declining kidney function partially mediating these relationships [127]. These predominantly observational data support PWV as a prognostic biomarker rather than an independent treatment indication [127].

### 7.3. Arterial Stiffness and Kidney Outcomes

The relationship between arterial stiffness and kidney dysfunction is bidirectional: excessive pulsatile energy may injure the renal microcirculation, while CKD-related inflammation, mineral dysregulation, and calcification accelerate arterial stiffening [122]. In the ARIC Study, among 3161 participants without CKD at baseline, 460 incident CKD events occurred during a median follow-up of 6.6 years. Participants in the highest cfPWV quartile had a 53% higher risk of incident CKD than those in the lowest quartile (HR 1.53, 95% CI 1.15–2.04), and higher cfPWV was also associated with a faster annual decline in eGFR [128,129]. In established CKD, non-invasive stiffness markers are also associated with composite renal outcomes, including eGFR decline, dialysis, and death [130]. Arterial stiffness may therefore provide information on renal risk before advanced CKD becomes clinically apparent.

### 7.4. Endothelial Dysfunction and Cardiovascular Outcomes

Flow-mediated dilation (FMD) independently predicts cardiovascular outcomes [131]. In a meta-analysis of 35 FMD studies including 17,280 participants, each 1% increase in FMD was associated with a 12% lower adjusted risk of cardiovascular events (RR 0.88, 95% CI 0.84–0.91). In the same analysis, a 0.1-unit increase in log-transformed reactive hyperemia index was associated with a 21% lower risk (RR 0.79, 95% CI 0.71–0.87) [125]. A separate meta-analysis of 23 prospective studies involving 14,753 participants found an overall RR of 0.92 (95% CI 0.88–0.95) for cardiovascular events per 1% higher FMD. The association was stronger in populations with established disease (RR 0.87, 95% CI 0.83–0.92) than in asymptomatic populations (RR 0.96, 95% CI 0.92–1.00) [132]. Both FMD and reactive hyperemia–peripheral arterial tonometry (RH-PAT) have predicted events in cardiovascular populations [29], although RH-PAT evidence remains less consistent because of device-specific methodology and heterogeneous thresholds [29].

Importantly, the incremental value of FMD is less robust in asymptomatic populations, where associations may weaken after adjustment for conventional risk factors and improvements in discrimination are often small [133]. Thus, endothelial dysfunction provides prognostic information but is less established than cfPWV for routine risk refinement.

### 7.5. Microvascular Dysfunction and Emerging Biomarkers

Microvascular phenotyping may capture vascular injury not reflected by large-artery stiffness or brachial FMD. Retinal vascular abnormalities, including altered vessel caliber and impaired dynamic vasodilation, have been associated with cardiovascular outcomes, while dynamic retinal assessment and AI-derived retinal phenotypes are emerging as potential risk-stratification tools [134,135,136,137]. In a systematic review and meta-analysis of 32 longitudinal studies including 105,845 participants, high versus normal CAVI was associated with a 46% higher risk of cardiovascular events (HR 1.46, 95% CI 1.22–1.75), while each SD/unit increase was associated with a 30% higher risk (HR 1.30, 95% CI 1.20–1.41). High CAVI was also associated with kidney-function decline (HR 1.30, 95% CI 1.18–1.43), whereas the association with mortality was not statistically significant (HR 1.31, 95% CI 0.92–1.87) [138].

However, prospective outcome evidence remains limited, methodological standardization is incomplete, and many studies are cross-sectional. These approaches therefore remain complementary or investigational rather than established prognostic tools [137].

### 7.6. Incremental Prognostic Value and Clinical Relevance

Prognostic association should be distinguished from clinical utility. Because conventional variables—including age, blood pressure, diabetes, lipids, smoking, eGFR, albuminuria, and established cardiovascular disease—already provide substantial prognostic information, vascular measures should demonstrate incremental discrimination, calibration, reclassification, and ideally improved clinical outcomes.

Arterial stiffness currently has the strongest evidence for incremental prediction, particularly in intermediate-risk populations; endothelial-function testing is less consistent, while microvascular and glycocalyx approaches remain insufficiently validated for routine prognostic use [3,122,137]. Importantly, evidence that vascular phenotype-guided treatment improves cardiovascular events, mortality, or CKD progression remains scarce.

### 7.7. Prognostic Interpretation Across the CKM Syndrome

Across the CKM continuum, cfPWV/aortic PWV remains the best-validated functional vascular marker for cardiovascular and renal prognosis, whereas FMD provides complementary endothelial information and microvascular techniques remain less mature [3,4,124,125,126,127,128,137]. The clinical relevance of these phenotypes increases with CKM burden, but their principal role is to complement rather than replace established risk assessment.

## 8. Multimodal Risk Models and Integration Frameworks

The multidimensional nature of cardiovascular–kidney–metabolic (CKM) syndrome supports a shift from isolated biomarkers toward integrated risk assessment. The 2026 AHA/ACC/ADA/ASN CKM guideline recommends CKM staging together with quantitative cardiovascular risk assessment using the PREVENT equations, which estimate ASCVD, heart failure, and total cardiovascular disease risk while incorporating variables relevant to the CKM spectrum, including kidney and metabolic factors [2]. Contemporary risk assessment therefore provides an appropriate clinical framework onto which vascular phenotyping may potentially be added.

Arterial stiffness and endothelial function capture complementary aspects of vascular injury. Endothelial dysfunction may reflect relatively early functional impairment related to metabolic abnormalities, inflammation, and oxidative stress, whereas increased arterial stiffness progressively reflects both functional changes and structural arterial remodeling. Both impaired flow-mediated dilation (FMD) and increased pulse wave velocity (PWV) have been independently associated with adverse cardiovascular outcomes [125,131,136]. Their complementary assessment may therefore provide a broader characterization of vascular dysfunction, although the incremental prognostic value of joint testing remains insufficiently established. A multimodal CKM risk framework could therefore integrate clinical risk and CKM stage, vascular function (FMD/PWV), structural vascular disease (e.g., CAC or carotid plaque), and cardiac and renal biomarkers. Such an approach may be particularly useful in patients in whom conventional risk estimates appear discordant with the observed vascular phenotype. The recently proposed Calculate–Personalize–Reclassify (C-P-R) framework similarly emphasizes sequential risk refinement, beginning with PREVENT-based estimation and followed, when appropriate, by risk enhancers, biomarkers, or imaging for detection of subclinical cardiovascular disease.

At present, however, FMD and PWV are not incorporated into PREVENT or recommended as routine CKM risk-reclassification tools. Their clinical value therefore remains complementary and requires prospective validation demonstrating incremental discrimination, reclassification, cost-effectiveness, and, importantly, improvement in therapeutic decision-making. Future multimodal models incorporating vascular measurements with imaging, biomarkers, longitudinal data, and machine-learning approaches may enable more individualized characterization of CKM-related vascular risk and identify distinct trajectories from early vascular dysfunction to overt cardiovascular disease.

## 9. Clinical Implementation Based on CKM Stages

The clinical implications discussed in this section are supported by different levels of evidence. CKM staging, PREVENT-based risk estimation, assessment of kidney markers, and guideline-directed preventive and cardiorenal therapies are derived from contemporary clinical guidelines and, where applicable, randomized outcome trials. In contrast, the stage-specific use of cfPWV, FMD, microvascular testing, and multimodal vascular phenotyping represents an interpretative framework proposed by the authors, because randomized trials demonstrating improved outcomes with vascular phenotype-guided management are currently lacking.

The clinical implementation of vascular phenotyping in cardiovascular–kidney–metabolic (CKM) syndrome should be stage-specific, risk-adapted, and integrated with conventional metabolic, renal, and cardiovascular assessment. The 2026 AHA/ACC/ADA/ASN CKM guideline conceptualizes CKM as a continuum from stage 0, characterized by the absence of recognized CKM risk factors, to stage 4, defined by established cardiovascular disease (CVD) in the presence of CKM risk factors [2]. CKM staging incorporates metabolic risk factors, kidney function, and CVD status, with combined assessment of estimated glomerular filtration rate (eGFR) and urinary albumin-to-creatinine ratio (UACR) recommended from stage 2 onward [2]. Albuminuria is particularly relevant because it reflects both kidney injury and systemic vascular dysfunction [2,139].

In adults aged 30–79 years without established CVD, the PREVENT equations provide 10- and 30-year estimates of ASCVD, heart failure (HF), and total CVD risk [2]. A predicted 10-year total CVD risk ≥20% constitutes a stage 3 criterion [1]. The AHA/ACC Calculate–Personalize–Reclassify (C-P-R) framework further emphasizes selective additional testing when it can meaningfully refine risk classification and influence management [140]. Accordingly, vascular phenotyping should not be applied as universal screening but as a complementary extension of risk-based assessment, tailored to CKM stage, baseline risk, clinical uncertainty, and the likelihood that the result will alter management.

### 9.1. CKM Stages 0–1: Prevention and Early Vascular Aging

CKM stage 0 denotes absence of recognized metabolic, renal, or cardiovascular risk factors, whereas stage 1 includes excess or dysfunctional adiposity without additional metabolic risk factors, CKD, or CVD [2]. The principal objective is primordial and primary prevention through surveillance and modification of adiposity, blood pressure, glycemia, lipids, kidney function, lifestyle, and other modifiable risk factors [2,140].

Selected individuals may nevertheless exhibit accelerated vascular aging before overt CVD. cfPWV may provide complementary information regarding early arterial remodeling, as arterial stiffness is associated with future cardiovascular events and may precede overt hypertension and CVD [3,141]. The CRAVE study further linked accumulation of cardiovascular risk factors to accelerated progression of cfPWV and augmentation index [142], while age-specific reference values may help identify stiffness disproportionate to chronological age [3].

Endothelial function may also identify early vascular abnormalities. FMD declines with aging and has been associated with cardiovascular events [141,143], whereas PAT offers an alternative assessment of reactive hyperemia but has less established incremental clinical value [144]. Importantly, population-based data indicate that neither FMD nor PAT substantially improves risk prediction beyond conventional factors in asymptomatic populations [145]. In our interpretation, based on the currently available evidence, CKM stages 0–1 are best suited to selective rather than universal vascular phenotyping: cfPWV may be useful when accelerated vascular aging is suspected, whereas FMD and PAT remain primarily specialized or research tools [3,141,142,143,144,145,146]. Abnormal findings may reinforce preventive strategies but should not independently trigger pharmacological treatment in the absence of validated thresholds.

### 9.2. CKM Stage 2: Metabolic Risk, Hypertension, Diabetes, or Early CKD

CKM stage 2 includes established metabolic or kidney abnormalities—including hypertension, hypertriglyceridemia, metabolic syndrome, type 2 diabetes, and moderate- to high-risk CKD—in the absence of subclinical or clinical CVD [2]. At this stage, vascular dysfunction and structural remodeling become increasingly relevant as CKM burden accumulates [1,3,147].

Assessment should include conventional metabolic variables, eGFR and UACR, together with PREVENT-based risk estimation where applicable [2]. A 10-year total CVD risk ≥7.5% may support prioritization of pharmacological risk-factor management, whereas ≥20% identifies a stage 3 risk-equivalent phenotype [2,140].

Arterial stiffness is particularly relevant because it integrates the cumulative effects of hypertension, diabetes, obesity, and declining kidney function. cfPWV is associated with cardiovascular events, HF, end-stage kidney disease, and mortality [5]. baPWV offers a more scalable alternative; cfPWV and baPWV correlate and show broadly similar associations with target-organ damage, although cfPWV may retain greater cardiovascular prognostic value [148,149,150]. Increased PWV may therefore identify vascular aging disproportionate to conventional risk-factor burden, while central pressure and wave-reflection indices provide complementary hemodynamic information [3].

Structural phenotyping may be useful when uncertainty regarding subclinical atherosclerosis persists. Carotid plaque is favored over routine cIMT as a risk-modifying measure [151,152,153], while CAC can reclassify selected patients with borderline or intermediate predicted risk and may identify low-risk individuals when CAC = 0 in appropriate clinical contexts [154,155]. CAC should nevertheless remain a decision-support tool rather than a universal screening test.

FMD, laser-based microvascular assessment, and retinal imaging may provide complementary mechanistic information, but their incremental value for routine risk prediction remains insufficiently established [152,156]. Based on the evidence reviewed above, we consider vascular phenotyping in stage 2 potentially useful for identifying accelerated vascular aging or subclinical structural disease and for integrating these findings with PREVENT estimates, metabolic risk, and kidney status in patients approaching a stage 3 phenotype.

### 9.3. CKM Stage 3: Subclinical Cardiovascular Disease or High Predicted Risk

CKM stage 3 marks the transition from metabolic and renal risk factors to demonstrable subclinical CVD or an equivalent level of predicted risk. It includes subclinical coronary atherosclerosis, pre-HF, very-high-risk CKD, or a PREVENT 10-year total CVD risk ≥20%, in the absence of clinical CVD [2]. Stage 3 criteria include CAC ≥100 Agatston units, moderate-to-severe incidental CAC, coronary atherosclerosis identified by coronary imaging, and low ankle-brachial index without symptomatic claudication [2,140]. Very-high-risk CKD and PREVENT risk ≥20% are likewise considered subclinical CVD risk equivalents [2,140].

Structural assessment therefore assumes greater clinical importance. CAC can substantially refine risk classification [2,140], while carotid plaque and ABI provide complementary evidence of systemic atherosclerotic disease, although their CKM-specific treatment thresholds are less established [2,140]. Importantly, CAC = 0 should not be interpreted as complete absence of cardiovascular risk in individuals with diabetes, smoking exposure, strong family history, or other high-risk features [2,140].

Functional vascular phenotyping may complement this structural information. Increased cfPWV is associated with cardiovascular risk and target-organ damage in metabolic syndrome and hypertension [157] and may identify disproportionate vascular aging in diabetes, obesity, and CKD. However, because cfPWV is not a formal stage 3 diagnostic criterion, it should remain complementary rather than determinative [2,140].

Pre-HF is another important stage 3 phenotype. Current guidance identifies pre-HF through elevated cardiac biomarkers and/or structural or functional cardiac abnormalities, with biomarker testing particularly relevant when 10-year PREVENT-HF risk is ≥5% [2,140]. Vascular measures may provide additional insight into the arterial component linking metabolic and renal abnormalities with myocardial dysfunction, but echocardiography, cardiac biomarkers, kidney function, UACR, and validated measures of atherosclerotic burden remain the principal clinical tools [2,140].

The higher absolute risk at stage 3 justifies more intensive evidence-based prevention. Contemporary CKM-directed therapies, including SGLT2 inhibitors in appropriately selected patients with diabetes or CKD, provide important cardiovascular and cardiorenal benefits [2,158,159,160]. Vascular phenotyping should therefore function primarily as a risk-refinement tool integrated with validated risk equations, imaging, kidney status, and cardiac assessment rather than as an independent therapeutic target.

### 9.4. CKM Stage 4: Established Cardiovascular Disease With or Without Kidney Failure

CKM stage 4 comprises established clinical CVD—including ASCVD, HF, stroke, peripheral artery disease (PAD), or atrial fibrillation—in individuals with CKM risk factors [2]. Stage 4a denotes absence of kidney failure, whereas stage 4b includes kidney failure, defined by eGFR <15 mL/min/1.73 m^2^ or chronic kidney replacement therapy [2,140]. At this stage, the principal objective shifts from detecting occult disease toward secondary prevention, disease-specific management, and characterization of residual risk.

Arterial stiffness and endothelial function may provide complementary information regarding residual vascular burden. cfPWV remains associated with cardiovascular risk but is limited by methodological and implementation considerations [141,143,151], while FMD may retain prognostic relevance particularly in populations with established disease [29,143]. Neither should replace established secondary-prevention strategies. Carotid imaging and assessment of the peripheral circulation may be useful when the distribution or extent of multibed vascular disease is clinically relevant [161,162,163].

PAD illustrates the importance of question-driven vascular assessment. Contemporary guidelines support anatomic imaging when it is expected to guide revascularization or otherwise modify management, rather than simply to document disease burden [161]. This principle is broadly applicable to stage 4 vascular phenotyping.

Kidney failure adds further complexity. Stage 4b is associated with pronounced arterial stiffness, medial vascular calcification, HF, and volume-related hemodynamic abnormalities [2,164,165]. Interpretation of vascular measurements and biomarkers may be more difficult at very low eGFR, and evidence for several therapies remains less complete because advanced kidney failure has historically been underrepresented in clinical trials [2]. Management therefore requires individualized, multidisciplinary decision-making.

Similarly, HF represents a multidimensional stage 4 phenotype in which vascular, cardiac, metabolic, and renal abnormalities coexist. Vascular parameters should be interpreted together with cardiac imaging, natriuretic peptides, kidney function, volume status, and functional capacity. Guideline-directed HF therapy and comprehensive CKM risk-factor management remain central, with SGLT2 inhibitors providing cardiovascular and kidney benefits across broad HF and CKD populations [158,159,166].

In our proposed framework, vascular phenotyping in stage 4 should be question-driven rather than protocol-driven, with the principal aim of characterizing residual vascular burden, multibed disease, and potentially treatment-relevant vascular abnormalities.

### 9.5. Proposed Clinical Algorithm: An Authors’ Integrative Framework

**Figure 2:**
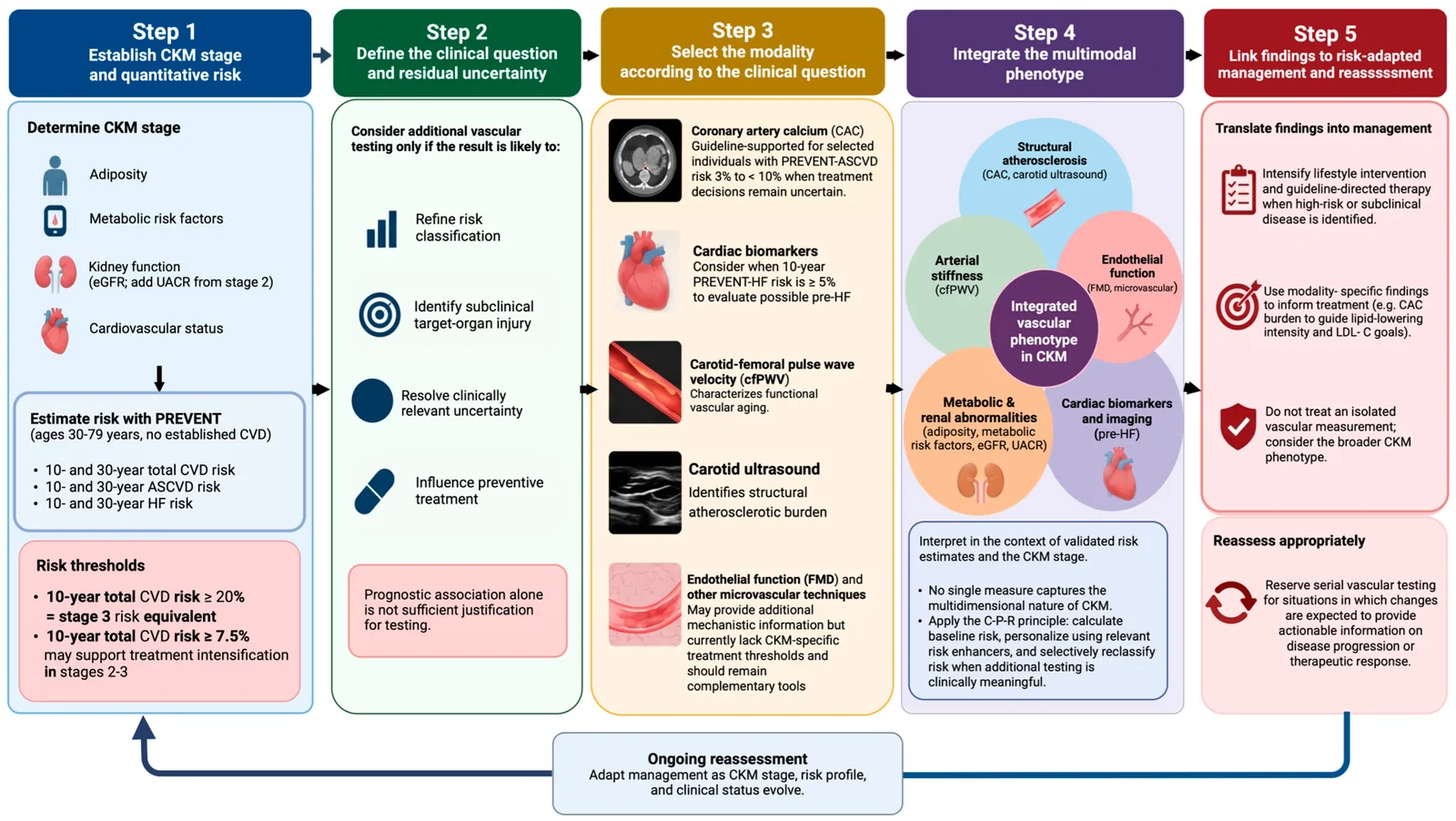
Proposed integrative framework for vascular phenotyping across the CKM continuum—Created in BioRender. Vladuta, T. (2026) https://BioRender.com/ggwjimb. The framework combines CKM staging and PREVENT-based quantitative risk assessment with definition of the clinical question, selective choice of vascular assessment modality, multimodal interpretation, and risk-adapted management with reassessment. Guideline-supported components include CKM staging, PREVENT-based risk estimation, eGFR/UACR assessment, CAC in selected patients, and cardiac biomarkers, whereas cfPWV, FMD, and other endothelial or microvascular techniques are presented as complementary phenotyping tools. This figure represents the authors’ integrative framework rather than a formally validated or guideline-endorsed algorithm. Abbreviations: ASCVD, atherosclerotic cardiovascular disease; CAC, coronary artery calcium; cfPWV, carotid–femoral pulse wave velocity; CKM, cardiovascular–kidney–metabolic; CVD, cardiovascular disease; eGFR, estimated glomerular filtration rate; FMD, flow-mediated dilation; HF, heart failure; LDL-C, low-density lipoprotein cholesterol; PREVENT, Predicting Risk of Cardiovascular Disease Events; UACR, urinary albumin-to-creatinine ratio.

Based on the evidence reviewed above, we propose an integrative framework for vascular phenotyping in CKM that combines CKM staging, PREVENT-based quantitative risk assessment, clarification of the clinical question, selective use of vascular modalities, multimodal interpretation, and risk-adapted management (Figure 2). CKM stage should be established from adiposity, metabolic risk factors, kidney function, and cardiovascular status, with eGFR and UACR incorporated as appropriate and PREVENT estimates used for quantitative cardiovascular risk assessment [2,140,167]. The expected vascular phenotype, potentially disproportionate findings, and the current role of individual vascular tests across CKM stages are summarized in Table 2.

Additional vascular testing should be considered only when it is likely to refine risk classification, identify subclinical target-organ injury, resolve clinically relevant uncertainty, or influence management [140]. Structural markers such as CAC and carotid imaging currently have the strongest role in selected risk-reclassification settings, whereas cfPWV may provide complementary information on vascular aging and FMD or other endothelial and microvascular techniques remain complementary because CKM-specific treatment thresholds are lacking [2,140,141].

Vascular findings should be interpreted within the broader CKM phenotype rather than in isolation, integrating validated risk estimates, metabolic and renal abnormalities, cardiac biomarkers, and structural imaging. Management should remain guideline-directed and risk-adapted, with serial vascular testing reserved for situations in which reassessment is expected to provide actionable information [2,121,140,168]. Prospective studies are still required to determine whether this integrated approach improves treatment allocation and clinical outcomes.

## 10. Limitations, Challenges and Future Directions

Several limitations currently restrict the translation of vascular phenotyping into routine CKM care. First, substantial methodological heterogeneity persists across vascular techniques. PWV values are influenced by measurement pathway, device, blood pressure, and age, whereas FMD remains highly dependent on acquisition conditions, operator expertise, and analytical methodology. Endothelial, microvascular, and structural techniques also assess different vascular beds and biological processes and should therefore not be considered interchangeable [3,4,146]. The lack of universally accepted thresholds for several measures further complicates comparison across studies and limits their use for individual clinical decision-making.

Second, much of the available evidence has been derived from selected cardiovascular, metabolic, or CKD populations rather than cohorts explicitly characterized across the contemporary CKM staging framework. Although several vascular markers are independently associated with cardiovascular and renal outcomes, prognostic association does not necessarily translate into incremental clinical utility. In particular, evidence that vascular phenotyping improves prediction beyond PREVENT, eGFR, UACR, established biomarkers, and validated structural imaging remains incomplete, and few studies have evaluated whether phenotype-guided therapeutic strategies improve cardiovascular events, CKD progression, or mortality [2,138,140,143].

Future research should therefore prioritize prospective, longitudinal studies encompassing the full CKM spectrum and using standardized vascular protocols. These studies should determine whether combinations of functional and structural vascular phenotypes improve discrimination, calibration, and clinically meaningful risk reclassification beyond contemporary CKM risk models. Particular attention should be given to individuals with discordance between predicted risk and observed vascular damage and to the transition from CKM stage 2 to stage 3, where additional phenotypic information may be most actionable. The prognostic value of serial changes in arterial stiffness or endothelial function and their responsiveness to CKM-directed therapies also require further evaluation.

Finally, multimodal approaches integrating vascular measurements with clinical variables, renal and cardiac biomarkers, imaging, and longitudinal data may provide a more individualized representation of CKM-related vascular injury. Machine-learning methods may facilitate integration of these heterogeneous data, but future models will require rigorous external validation, assessment of cost-effectiveness, and demonstration that improved prediction translates into better treatment allocation and patient outcomes. Until such evidence is available, vascular phenotyping should complement rather than replace established CKM staging and guideline-based risk assessment.

## 11. Conclusions

Vascular dysfunction represents an important link between metabolic abnormalities, kidney disease, and cardiovascular injury across the CKM continuum. Endothelial dysfunction, arterial stiffness, microvascular abnormalities, and structural vascular disease capture complementary stages and dimensions of this process, ranging from potentially reversible functional impairment to established vascular remodeling and atherosclerotic burden.

Among currently available techniques, cfPWV has the strongest evidence as a reproducible marker of arterial stiffness and cardiovascular risk, whereas FMD provides complementary information on endothelial function but remains more technically demanding. CAC and vascular plaque imaging provide stronger evidence for clinical risk reclassification, while newer microvascular and endothelial approaches remain primarily investigational. Accordingly, vascular phenotyping should be viewed as an integrated, risk-adapted strategy rather than as the routine application of individual vascular tests. Its greatest potential lies in complementing CKM staging, PREVENT-based risk estimation, kidney and cardiac biomarkers, and structural imaging to identify individuals with disproportionate or subclinical vascular injury. Prospective studies are now required to determine whether such multimodal phenotyping can improve therapeutic decision-making and ultimately translate into better cardiovascular and kidney outcomes.

## Figures and Tables

**Table 1:** Summary of vascular phenotyping modalities across the CKM spectrum.

Modality	Vascular Phenotype Assessed	Main Strengths	Main Limitations	Potential Role in CKM
**Carotid–femoral PWV (cfPWV)**	Central arterial stiffness/vascular aging	Reference non-invasive measure of aortic stiffness—extensive validation; strong association with cardiovascular events, mortality, and kidney outcomes [32,34,35,36,37,38,39,121]	Requires standardized path-length measurement, dedicated equipment, and trained personnel; blood-pressure dependent	Best-established functional vascular phenotype; potentially useful for identifying accelerated vascular aging and refining risk, particularly in CKM stages 2–3
**Brachial–ankle PWV (baPWV)**	Combined central and peripheral arterial stiffness	Simple, reproducible, scalable; established prognostic associations [40,41,42,43,44,45]	Influenced by muscular arteries and peripheral resistance; not interchangeable with cfPWV	Practical alternative where cfPWV is unavailable
**Estimated PWV (ePWV)**	Expected age- and BP-related arterial stiffness	No dedicated vascular equipment required; applicable to large populations; prognostic associations demonstrated [46,47,48,49,121]	Calculated rather than directly measured; limited ability to detect individual deviation from expected stiffness	Epidemiological risk characterization rather than individualized vascular phenotyping
**Augmentation index/central BP**	Wave reflection and central hemodynamic load	Provides information complementary to PWV and reflects ventricular–arterial interaction [1,39,50,51,52,53,54,55,56,57,58,59,60,61]	Strongly influenced by heart rate, height, age, sex, and peripheral vascular tone	Complementary characterization of the hemodynamic consequences of arterial stiffening
**Flow-mediated dilation (FMD)**	Conduit-artery endothelial function	Most established non-invasive endothelial test; mechanistically informative; associated with cardiovascular outcomes [4,6,67,68,69,70,71,72,73,74]	Operator- and protocol-dependent; substantial biological and technical variability; inconsistent incremental prediction	Potentially informative across early or intermediate CKM but not suitable for universal screening
**Peripheral arterial tonometry/RHI**	Predominantly digital microvascular reactivity	Largely operator-independent and technically simpler than FMD [66,75,76,77,78,79,80,81,82]	Uncertain specificity for NO-mediated dysfunction; heterogeneous thresholds and prognostic results	Complementary endothelial/microvascular phenotyping, predominantly in specialized or research settings
**Laser Doppler/laser speckle imaging**	Cutaneous microvascular perfusion and reactivity	Non-invasive assessment of dynamic microvascular responses; LSCI provides high temporal and spatial resolution [83,84,85,86,87]	Sensitive to environmental conditions and spatial heterogeneity; limited standardization and clinical outcome evidence	Research-oriented characterization of early microvascular dysfunction
**Sublingual glycocalyx imaging**	Endothelial glycocalyx/microvascular barrier integrity	Biologically attractive marker of early endothelial injury; rapid, non-invasive assessment [88,89,90,91]	Operator dependence, reproducibility concerns, and inconsistent association with clinical CVD [91,92]	Investigational—insufficient evidence for routine CKM risk stratification
**Carotid/femoral plaque ultrasound**	Structural atherosclerotic burden	Inexpensive, radiation-free; plaque burden predicts events better than cIMT and may improve risk classification [96,97,98,99,100,101,102,103,104,105,106,107]	Operator dependence and heterogeneity in plaque quantification protocols	Clinically relevant structural phenotype, particularly when conventional risk assessment remains uncertain
**Coronary artery calcium (CAC)**	Coronary atherosclerotic burden	Strongest evidence for incremental ASCVD risk reclassification; established role in contemporary prevention frameworks [97,108,109,110,111,112,113]	Ionizing radiation; does not distinguish intimal from medial calcification, particularly relevant in CKD and diabetes	Most clinically established structural risk-reclassification tool—particularly relevant to transition toward CKM stage 3

**Note:** The vascular phenotypes presented are not mutually exclusive; multiple functional and structural abnormalities may coexist within the same patient and collectively define the overall vascular phenotype. **Abbreviations:** baPWV, brachial–ankle pulse wave velocity; BP, blood pressure; CAC, coronary artery calcium; cfPWV, carotid–femoral pulse wave velocity; CKM, cardiovascular–kidney–metabolic; FMD, flow-mediated dilation; LSCI, laser speckle contrast imaging; NO, nitric oxide; PWV, pulse wave velocity; RHI, reactive hyperemia index.

**Table 2:** Potential role of vascular phenotyping across CKM stages.

CKM Stage	Expected Vascular Phenotype	Disproportionate/Discordant Finding	Most Informative Markers	Current Testing Role
**0–1**	Predominantly preserved vascular structure- functional abnormalities may be subtle or absent	Markedly increased cfPWV for age/BP—impaired endothelial function; definite plaque/CAC despite low clinical burden	cfPWV, selected FMD/PAT	Selective; endothelial/microvascular testing mainly research/specialized
**2**	Increasing arterial stiffness and endothelial dysfunction may emerge—structural disease may still be absent	Marked cfPWV elevation or definite plaque/CAC disproportionate to conventional risk burden	cfPWV, carotid plaque, CAC when risk is uncertain	Selective risk refinement
**3**	Structural disease or high-risk phenotype becomes dominant- functional abnormalities may coexist	Functional vascular injury substantially exceeding structural burden, or extensive multibed disease	CAC, plaque/ABI, cfPWV complementary	Structural testing clinically relevant; functional testing complementary
**4**	Established structural and functional vascular abnormalities frequently coexist	Disproportionate residual or multibed vascular burden relevant to management	Question-driven imaging, cfPWV/FMD only when clinically informative	Question-driven; not for reclassification

**Note:** These stage-specific expectations are qualitative and should not be interpreted as validated reference ranges. Quantitative CKM stage-specific thresholds are currently unavailable for most functional and microvascular measures. Vascular abnormalities may coexist within the same patient, and discordance refers to vascular damage that appears disproportionate to the overall CKM stage or conventional risk burden rather than to a formally validated criterion for stage reclassification.

## Data Availability

No new data were created or analyzed in this study. Data sharing is not applicable to this article.

## Abbreviations

The following abbreviations are used in this manuscript:

AAC	Abdominal aortic calcification
ABI	Ankle–brachial index
ACC	American College of Cardiology
ADA	American Diabetes Association
AHA	American Heart Association
AIx	Augmentation index
aPWV	Aortic pulse wave velocity
ARIC	Atherosclerosis Risk in Communities
ASCVD	Atherosclerotic cardiovascular disease
ASN	American Society of Nephrology
baPWV	Brachial–ankle pulse wave velocity
BMI	Body mass index
BP	Blood pressure
CAC	Coronary artery calcium
CAD	Coronary artery disease
CAVI	Cardio-ankle vascular index
cfPWV	Carotid–femoral pulse wave velocity
CKD	Chronic kidney disease
CKD-	Chronic kidney disease–mineral and bone disorder
CKM	Cardiovascular–kidney–metabolic
C-P-R	Calculate–Personalize–Reclassify
CRIC	Chronic Renal Insufficiency Cohort
CVD	Cardiovascular disease
eGFR	Estimated glomerular filtration rate
EndMT	Endothelial-to-mesenchymal transition
eNOS	Endothelial nitric oxide synthase
ePWV	Estimated pulse wave velocity
ESC	European Society of Cardiology
FMD	Flow-mediated dilation
HF	Heart failure
HFpEF	Heart failure with preserved ejection fraction
hs-cTnI	High-sensitivity cardiac troponin I
hs-cTnT	High-sensitivity cardiac troponin T
LDF	Laser Doppler flowmetry
LDPI	Laser Doppler perfusion imaging
LSCI	Laser speckle contrast imaging
MAP	Mean arterial pressure
NO	Nitric oxide
NT-proBNP	N-terminal pro-B-type natriuretic peptide
OCT	Optical coherence tomography
PAD	Peripheral artery disease
PAI-1	Plasminogen activator inhibitor-1
PAT	Peripheral arterial tonometry
PBR	Perfused boundary region
PI3K	Phosphoinositide 3-kinase
PVAT	Perivascular adipose tissue
PWV	Pulse wave velocity
RAAS	Renin–angiotensin–aldosterone system
RBC	Red blood cell
RBP4	Retinol-binding protein 4
RHI	Reactive hyperemia index
RH-PAT	Reactive hyperemia–peripheral arterial tonometry
ROS	Reactive oxygen species
SDF	Sidestream dark-field
SGLT2	Sodium–glucose cotransporter 2
TMAO	Trimethylamine N-oxide
UACR	Urinary albumin-to-creatinine ratio
VCAM-1	Vascular cell adhesion molecule-1
VSMC	Vascular smooth muscle cell
